# Combining carbon ion irradiation and non-homologous end-joining repair inhibitor NU7026 efficiently kills cancer cells

**DOI:** 10.1186/s13014-015-0536-z

**Published:** 2015-11-09

**Authors:** Hongyu Ma, Akihisa Takahashi, Yukari Yoshida, Akiko Adachi, Tatsuaki Kanai, Tatsuya Ohno, Takashi Nakano

**Affiliations:** Department of Radiation Oncology, Gunma University Graduate School of Medicine, 3-39-22 Showa-machi, Maebashi, 371-8511 Gunma Japan; Gunma University Heavy Ion Medical Center, 3-39-22 Showa-machi, Maebashi, 371-8511 Gunma Japan

**Keywords:** Carbon ion irradiation, DNA double-strand breaks, Homologous recombination repair, Non-homologous end-joining repair, Radiosensitization

## Abstract

**Background:**

Our previous data demonstrated that targeting non-homologous end-joining repair (NHEJR) yields a higher radiosensitivity than targeting homologous recombination repair (HRR) to heavy ions using DNA repair gene knockouts (KO) in mouse embryonic fibroblast (MEF). In this study, we determined if combining the use of an NHEJR inhibitor with carbon (C) ion irradiation was more efficient in killing human cancer cells compared with only targeting a HRR inhibitor.

**Methods:**

The *TP53*-null human non-small cell lung cancer cell line H1299 was used for testing the radiosensitizing effect of NHEJR-related DNA-dependent protein kinase (DNA-PK) inhibitor NU7026, HRR-related Rad51 inhibitor B02, or both to C ion irradiation using colony forming assays. The mechanism underlying the inhibitor radiosensitization was determined by flow cytometry after H2AX phosphorylation staining. HRR-related *Rad54*-KO, NHEJR-related *Lig4*-KO, and wild-type *TP53*-KO MEF were also included to confirm the suppressing effect specificity of these inhibitors.

**Results:**

NU7026 showed significant sensitizing effect to C ion irradiation in a concentration-dependent manner. In contrast, B02 showed a slight sensitizing effect to C ion irradiation. The addition of NU7026 significantly increased H2AX phosphorylation after C ion and x-ray irradiations in H1299 cells, but not B02. NU7026 had no effect on radiosensitivity to *Lig4*-KO MEF and B02 had no effect on radiosensitivity to *Rad54*-KO MEF in both irradiations.

**Conclusion:**

These results suggest that inhibitors targeting the NHEJR pathway could significantly enhance radiosensitivity of human cancer cells to C ion irradiation, rather than targeting the HRR pathway.

**Electronic supplementary material:**

The online version of this article (doi:10.1186/s13014-015-0536-z) contains supplementary material, which is available to authorized users.

## Background

Recently, carbon (C) ion radiotherapy has become an increasingly available option for the treatment of various malignancies due to a superior dose distribution and a high relative biological effectiveness (RBE) [1–4]. C ion irradiation can induce a variety of DNA toxic lesions including double-strand breaks (DSBs), which are the most lethal as an accumulation of misrepaired or unrepaired DSBs can lead to a massive loss of genetic information and cell death [5–7]. However, there are two major pathways in mammalian cells to repair a DSB: non-homologous end-joining repair (NHEJR) and homologous recombination repair (HRR) [8, 9]. Heavy ion irradiations induce more complex DSB damages and/or clustered damages around the break sites [10, 11] and are often repaired less efficiently compared with traditional x-ray radiotherapy [12]. Although recent work from our laboratory demonstrated that the repair of DSBs induced by heavy ions is slower than those induced by irradiation with x-rays [13], it is believed that these DSB repairs also contribute to radiation resistance in human cancer cells. Therefore, it is essential to identify new targets in DSB repair pathways and develop inhibitors to enhance the effect of C ion radiotherapy.

NHEJR is an error-prone and “quick” process compared with HRR, often associated with small insertions or deletions at the repaired break site [8, 14, 15] and is the dominant DSB repair pathway throughout the cell cycle [16, 17]. In contrast, HRR is an error-free repair pathway and only occurs in the late S/G_2_ phase of the cell cycle when the sister chromatid is in close proximity. This is because HR repair requires undamaged homologous DNA sequences as the repair template to rejoin the broken ends precisely [9, 14, 15].

The catalytic subunit of DNA-dependent protein kinase (DNA-PKcs) plays a critical role in the NHEJR pathway [18, 19]. After end recognition by the Ku70/Ku80 heterodimer, DNA-PKcs is rapidly recruited to the DSB damages and is phosphorylated at the Thr2609 and Ser2056 clusters by ataxia telangiectasia mutated (ATM) and itself, respectively [20, 21]. The recruitment and phosphorylation of DNA-PKcs contributes to processing and direct ligation of broken DNA ends by ligase 4 (Lig4) [14]. In response to DSB damages, Rad51 family proteins are recruited to the nucleoprotein filaments by Mre11-Rad50-Nbs1 (MRN) complex, as specific HRR proteins for homologous pairing and strand transfer of DNA. In addition, several studies have reported that DNA-PK inhibitors have potential to enhance the radiation sensitivity to photon beams in different tumors such as colon, breast, and prostate cancer [22–24]. Our previous data showed that targeting NHEJR yielded a higher radiosensitivity than targeting HRR to heavy ion irradiations using *TP53-*null DNA repair gene knock-out (KO) mouse embryonic fibroblast (MEF) cell lines [25]. However, little is known about these DNA repair inhibitors as related to the effects of C ion irradiation in human cancer cells.

This study aims to assess the combined effects of C ion irradiation with the DNA repair inhibitors on cell killing in human cancer cells to determine the primary target for further enhancement of the effects of C ion radiotherapy and improvement on existing therapeutic strategies. Therefore, we studied the sensitizing effect of NHEJR and HRR inhibitors to C ion irradiation in H1299, A172, U251MG, and *TP53-*null DNA repair gene KO MEF.

## Methods

### Cells

Human non-small cell lung cancer (NSCLC) H1299 (*TP53*-null) cells (population doubling time was 17 h) were provided by Dr. Moshe Oren (Weizmann Institute of Science, Rehovot, Israel). *TP53*-KO MEF (population doubling time was 12 h) *Lig4*^+^/_+_*Rad54*^−^/_−_ (*Rad54*-KO), *Lig4*^−^/_−_*Rad54*^+^/_+_ (*Lig4*-KO), and *Lig4*^+^/_+_*Rad54*^+^/_+_ (wild-type) were provided by Dr. Frederick W. Alt (Harvard Medical School, Boston, MA). Human glioblastoma A172 (population doubling time was 31 h) (wild-type *TP53*: wt*p53*) and U251MG (population doubling time was 21 h) (mutated *TP53*: m*p53*) were purchased from ATCC (VA, USA). All cells were cultured in Dulbecco’s modified Eagle’s medium (D-MEM) (Wako, Osaka, Japan) with high glucose and L-glutamine and supplemented with 10 % heat-inactivated fetal bovine serum (FBS), penicillin (100 U/ml), streptomycin (100 μg/ml) and HEPES (10 mM) at 37 °C in a humidified atmosphere of 5 % CO_2_.

### Irradiations

Exponentially growing cells were irradiated with C ions or x-rays. X-ray irradiations were completed using a 200-kVp x-ray generator (TITAN-225S, Shimadzu, Kyoto, Japan), operated at 14.6 mA with a total filtration of 0.5-mm Aluminum plus 0.5-mm Copper or using a 150-kVp x-ray generator (MBR-1520R-4, Hitachi, Tokyo, Japan), operated at 20 mA, with a 0.5-mm Aluminum plus 0.3-mm Copper filtration. The x-ray dose rate was about 1.3 Gy/min and 1 Gy/min, respectively. C ion irradiations were performed at the Gunma University Heavy Ion Medical Center using the same beam specifications used in clinical settings (energy of 290 MeV/nucleon and a dose-averaged linear energy transfer (LET) of approximately 50 keV/μm at the center of the spread-out Bragg peak (SOBP)). Monolayer cells were placed in the center of the SOBP with a length of 6 cm [26]. The cells used for DNA damage analysis were irradiated separately from cells used for colony forming assays.

### Treatments

**Fig. 1:**
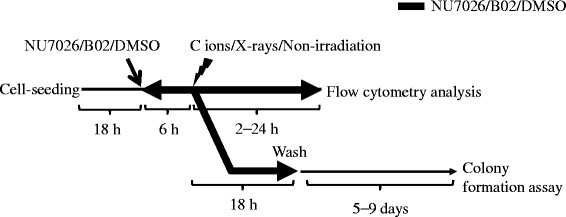
Schematic work flow for experiments

Cells were treated with either the DNA-PK inhibitor NU7026 (Calbiochem, Darmstadt, Germany), Rad51 inhibitor B02 (Calbiochem), or a vehicle (dimethyl sulfoxide (DMSO)) control beginning 6 h prior to irradiation. All inhibitors were removed 18 h after irradiation and the cells were cultured with fresh medium after irradiation for colony forming assays, but not for flow cytometry analysis (Fig. 1).

### Colony forming assays

Cell survival was defined using a standard colony forming assay. Cells were trypsinized and seeded into T-25 flasks at defined densities 24 h before irradiation. After the cells were transferred to fresh media, at about 5 days for MEF, 7 days for H1299 cells, 6–9 days for U251 and A172 cells, the surviving colonies were fixed with methanol and stained with 5 % Giemsa solution (Fig. 1). The number of colonies containing at least 50 cells were counted and surviving fractions (SF) were calculated from the number of colonies formed in the irradiated flasks compared with the number formed in the un-irradiated control, in which the plating efficiency (PE) was defined as the percentage of cells plated that form colonies in un-irradiated flasks. Specifically, the SF was defined as the number of colonies formed, divided by the number of cells plated multiplied by the PE. Each point in the corresponding figures represents the mean surviving fraction calculated from three independent experiments done in triplicate for each treatment condition, and the error bars represent the standard deviation (SD). A sensitization ratio (SR) for the inhibitors was calculated to quantify differences between survival curves. The SR was defined as the radiation dose resulting in a 10 % survival rate divided by the radiation dose resulting in a 10 % survival rate for inhibitor-treated cells.

### DNA damage analysis

After treatment as described above, H2AX phosphorylation was analyzed in cells derived from monolayer cultures following incubation with trypsin-EDTA. Cells were pelleted by centrifugation, then fixed in 70 % ethanol and stored at −20 °C. Fixed cells were washed one time with phosphate-buffered saline (PBS) containing 1 % bovine serum albumin (BSA) and blocked with 10 % horse serum and 0.5 % Tween20 in PBS at room temperature for 15 min. Cells were incubated with a mouse monoclonal antibody against human γH2AX (1:300; Millipore, Billerica, MA) in PBS with 1 % BSA and 0.5 % Tween20 at room temperature for 1 h. After one wash with PBS with 1 % BSA, cells were incubated with an Alexa Fluor 488-conjugated goat anti-mouse IgG antibody (1:400; Molecular Probes) at room temperature for 1 h. Cells were washed two times and subsequently permeabilized with 400-μl PBS containing 0.1-mg/ml PI, 0.1 % Triton X-100, and 400-μl (0.8 mg) DNase-free RNaseA at room temperature in the dark for 30 min, then kept on ice. Samples were measured using a FACSCalibur (BD, Franklin Lakes, NJ). Data were analyzed with FlowJo software (Digital Biology, Tokyo, Japan).

### Statistical analysis

All values were expressed as the mean ± SD. Data were analyzed using a Student’s *t* test with a *p* < 0.05 considered as significant.

## Results

### Sensitizing effect of NU7026 and B02 to C ion and x-ray irradiations

**Fig. 2:**
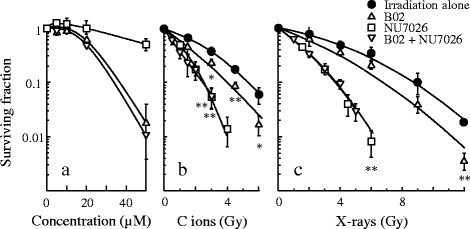
Survival curves after NU7026, B02 treatment and radiation exposure for human H1299 cells. NU7026 alone, B02 alone and both concentration-dependent changes (**a**). C ion irradiation dose-dependent changes (**b**) and the x-ray irradiation (**c**) when treated or not treated with 10 μM inhibitors. The presented results are the mean and SD of three independent experiments. Data were statistically evaluated with student’s *t* test with comparisons between irradiation alone and other treated groups (* *p* < 0.05; ** *p* < 0.01)

**Fig. 3:**
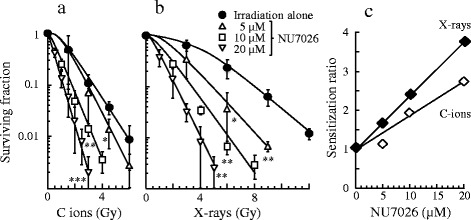
Survival curves after NU7026 and radiation exposure and sensitization ratio of NU7026 for human H1299 cells. C ion irradiation dose-dependent changes (**a**) and the x-rays (**b**) when treated or not treated with 5–20 μM NU7026. NU7026 concentration-dependent sensitization ratio values to C ion and x-ray irradiations (**c**). The presented results are the mean and SD of three independent experiments. Data were statistically evaluated with student’s *t* test with comparisons between irradiation alone and other treated groups (* *p* < 0.05; ** *p* < 0.01; *** *p* < 0.001)

Colony forming assays were conducted following 24 h exposure to graded concentrations of NU7026, B02 or two-drug combination. H1299 cells exposed to a NU7026 concentration of < 20 μM resulted in nearly no cytotoxicity, and the surviving fraction was approximately 90 %. The B02 and two-drug combination concentrations of < 10 μM resulted in minimal cytotoxicity (Fig. 2a). The independent cytotoxicity of NU7026 was lower than B02 in A172 cells and U251MG cells (Additional file 1: Fig. S1). To clarify if these inhibitors could enhance sensitivity to C ion irradiation, we examined the surviving fraction at a concentration of 10 μM and it did not show independent cytotoxicity. Exposure of H1299 cells to 10 μM B02 resulted in a slight increase in radiosensitivity to the C ion and x-ray irradiations with a SR of 1.34 and 1.21 (Table 1), respectively, at the 10 % survival dose (*D*_10_). H1299 cells exposed to 10 μM NU7026 enhanced the sensitivity of cells to both irradiations significantly. However, the two-drug combination did not show an additive effect (Fig. 2b and c). In addition, targeting NHEJR in A172 and U251MG cells greatly sensitized the cells to the C ion irradiation, but targeting HRR in these cells resulted in a slight change in the sensitivities, and the SR value of 10 μM NU7026 for A172 and U251MG cells at the *D*_10_ from the C ion irradiation was 1.87 and 1.52, respectively (Additional file 2: Fig. S2). Building on the results of our previous work on DNA repair-KO MEF, this study investigated graded concentrations of NU7026 to clarify if the sensitizing effect of NU7026 to C ion irradiation occurred in a concentration-dependent manner. NU7026-sensitized H1299 cells to the C ion and x-ray irradiations (Fig. 3a and b) in a concentration-dependent manner. Incidentally, the RBE of the C ion irradiation was 2.4 at the *D*_10_, indicating a stronger ability to induce DNA damage relative to x-rays. Additionally, although the SR value of NU7026 for H1299 cells at the *D*_10_ from C ion irradiation was slightly lower than the value for x-rays (about 66 % less), NU7026 dramatically increased the SR to both C ion and x-ray irradiations in a concentration-dependent manner (Fig. 3c) (Table 2), indicating that targeting NHEJR is more effective in enhancing the sensitivity of human tumor cells to C ion irradiation than HRR.

**Table 1:** *D*_10_ values and sensitization ratios for B02, NU7026, and combination of both in H1299 cells

	*D*_10_		Sensitization ratio	
	C ions	X-rays	C ions	X-rays
Irradiation alone	5.47 ± 0.35	8.83 ± 0.76	1.00	1.00
B02 (10 μM)	4.07 ± 0.23	7.28 ± 0.48	1.34	1.21
NU7026 (10 μM)	2.63 ± 0.31	3.77 ± 0.32	2.08	2.34
B02 + NU7026	2.47 ± 0.32	3.80 ± 0.26	2.21	2.32

*D*_10_ dose giving a survival of 10 %; C ions carbon ions

**Table 2:** *D*_10_ values and sensitization ratios for different concentrations of NU7026 in H1299 cells

NU7026 (μM)	*D*_10_		Sensitization ratio	
	C ions	X-rays	C ions	X-rays
0	3.27 ± 0.35	7.93 ± 0.45	1.00	1.00
5	2.60 ± 0.69	4.93 ± 0.67	1.26	1.61
10	1.67 ± 0.06	3.20 ± 0.10	1.96	2.48
20	1.20 ± 0.17	2.10 ± 0.53	2.73	3.78

*D*_10_ dose giving a survival of 10 %; C ions carbon ions

### DNA DSB repair ability in H1299 cells after exposure to NU7026, B02, and irradiation

**Fig. 4:**
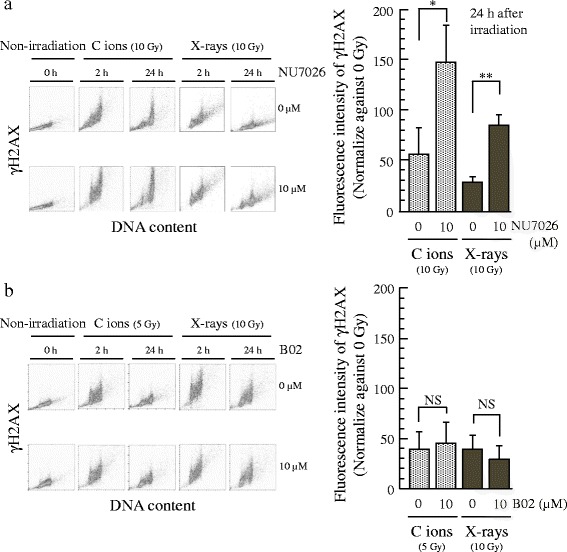
Effect of NU7026, B02 and irradiation on DNA damage accumulation in H1299 cells. Fluorescence intensity of γH2AX in 10 μM NU7026 (**a**), 10 μM B02 (**b**) and/or radiation-treated cells. The presented results are the mean and SD of three independent experiments. Data were statistically evaluated with student’s *t* test with comparisons between irradiation alone and other treated groups (* *p* < 0.05; ** *p* < 0.01; NS, non-significant)

Effects of NU7016, B02, and irradiation on DNA damage accumulation in H1299 cells was analyzed using flow cytometry after γH2AX staining. Histone γH2AX is an important marker protein for detecting DNA DSB expression. The combination of 10 μM NU7026 and irradiation with C ion or x-ray irradiation markedly increased the H2AX phosphorylation at 24 h after irradiation relative to irradiation alone (Fig. 4a); but the combination of B02 and irradiation did not affect the H2AX phosphorylation (Fig. 4b), indicating suppression of DNA repair ability and the accumulation of unrepaired lethal DNA damage in the cells after NU7026 treatment, but not B02.

### Radiosensitizing effect of NU7026 and B02 in MEF with a specific NHEJR-KO and HRR-KO

**Fig. 5:**
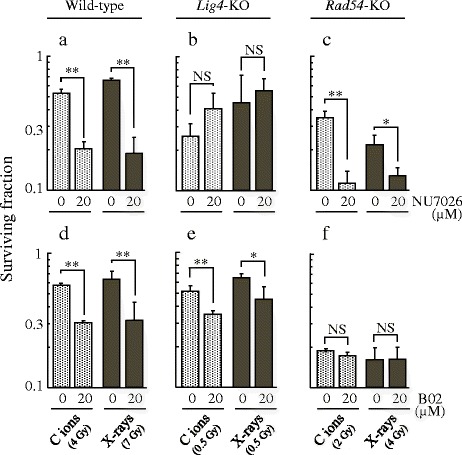
Survival fraction after radiation exposure with NU7026 or B02 treatment in MEF. 20 μM NU7026 treatment (**a**–**c**), 20 μM B02 treatment (**d**–**f**). Wild-type cells (**a** and **d**), *Lig4*-KO cells (**b** and **e**), *Rad54*-KO cells (**c** and **f**). The presented results are the mean and SD of three independent experiments. Data were statistically evaluated with the student’s *t* test with comparisons between irradiation alone and other treated groups (* *p* < 0.05; ** *p* < 0.01; NS, non-significant)

To confirm the suppressing specificity of these inhibitors, the radiosensitizing effect of NU7026 and B02 were analyzed using colony formation assays in HRR-related *Rad54*-KO, NHEJR-related *Lig4*-KO, and wild-type cells. Exposure of wild-type cells to 20 μM NU7026 and B02 resulted in an increase in radiosensitivity to C ion and x-ray irradiations. However, NU7026 had no effect on radiosensitivity of *Lig4*-KO cells and B02 had no effect on radiosensitivity of *Rad54*-KO cells in both irradiations (Fig. 5).

## Discussion

NHEJR is the dominant DSB repair pathway and is a more severe threat to survival than HRR exposed to low-LET radiation [15, 27]. However, the primary repair pathway for DSBs in high-LET heavy ion irradiations has remained elusive. In this study, cellular radiosensitivity to C ion and x-ray irradiations after treatment with inhibitors targeting NHEJR and HRR was determined using human cancer cells. This study demonstrated that NU7026 is more effective for enhancing the sensitivity of human tumor cells to C ion irradiation. In contrast, B02 did not demonstrate increased sensitivity at the concentration studied without independent cytotoxicity, and the two-drug combination showed no additive effect. These findings are consistent with our previous report using repair-KO MEF [25]. In addition, similar results have been recently reported in repair-KO Chinese hamster cell lines using proton and C ion irradiation [28]. The results of radiosensitizing effect of NHEJR inhibitor are also consistent with other studies involving low-LET irradiations [29–33]. Furthermore, this suggests that the major target leading to enhanced radiosensitivity of tumor cells to high-LET radiation is NHEJR rather than HRR, and similar to low-LET radiation. In contrast, HRR-deficient, but not NHEJR inhibited, tumor cells showed markedly increased radiosensitivity to low-LET proton irradiation accompanied with reduced phosphorylation of DNA-PKcs after proton irradiation compared with photon irradiation [34]. The reason for this difference may be related to the inherent differences between radiation quality from proton and C ion irradiation. In addition, our previous report demonstrated that NHEJR is the dominant target for sensitizing cells to C ion irradiation regardless of LET values using MEF cells [25]. NU7026-sensitized *TP53*-mull H1299 cells, wt*p53* A172 cells, and m*p53* U251MG cells to C ion irradiation. This suggests that the sensitizing effect of the NHEJR inhibitor to both irradiations is *TP53*-independent. The independent cytotoxicity of NU7026 was lower than B02. This suggests that administration of NU7026 at a concentration without systemic cytotoxicity to patients may be possible. Consequently, although the SR value of NU7026 for H1299 cells to the C ion irradiation at *D*_10_ was slightly lower than that of x-rays, NU7026 dramatically increased the SR to both irradiations in a concentration-dependent manner. It has been reported that NHEJR is suppressed by high-LET radiation, because high-LET radiation results in accumulation of short DNA fragments (<40 base pairs) along the beam track and these short fragments bind to Ku proteins, which are the sensor of DSBs in the NHEJR pathway [35–37]. Therefore, the SR of NU7026 to the C ion irradiation is slightly lower than that of x-rays.

In contrast, as NHEJR is suppressed by high-LET radiation and high-LET radiation-induced short fragments of linear DNA do not affect the HRR sensor MRE11 protein, it has been reported that the dominant repair pathway during high-LET radiation is HRR using DNA repair-KO Chinese hamster cells, MEFs, and chicken DT40 cells [35, 36]. Moreover, high-LET radiation-induced DSBs are too complex with dirty broken ends to repair efficiently by NHEJR [12]. However, NHEJR inhibition showed a high SR to C ion irradiation relative to HRR inhibition in human cancer cells. This point suggests that high-LET radiation only partly suppresses NHEJR despite the suppression of the Ku-dependent NHEJR pathway. High-LET radiation-induced short DNA fragments suppression in the Ku-dependent NHEJR pathway, but not in the PARP-1-dependent NHEJR pathway [35], in support of this suggestion. Moreover, a possible explanation for this difference may be that the proliferation ratio of Chinese hamster and chicken cells is higher than human cells because of the shorter doubling time compared with human cells [38]. This means that the contribution of HRR in these cells is higher than in human cells, because the late S/G_2_ phase is more populous. HRR can repair DSBs using the undamaged DNA homologue as a template in the late S/G_2_ phase.

DNA repair ability of H1299 cells after treatment with NHEJR inhibitor and HRR inhibitor was examined using flow cytometry. The combination of NU7026 and both irradiations markedly increased H2AX phosphorylation at 24 h after irradiation, but B02 did not affect the H2AX phosphorylation. This result suggests that the suppression effect of DNA repair capacity by NHEJR inhibition is stronger than HRR inhibition at a concentration without independent cytotoxicity. The DNA repair suppression effect of NHEJR inhibition in this study is consistent with similar studies involving photon low-LET irradiation [31, 32].

DNA-PKcs is a multifunction protein kinase, necessary for NHEJR, telomere regulation, and mitosis [39]. This study determined the specificity of the inhibitors of interest to DNA repair pathways using repair-KO MEF cells. NU7026 had no effect on radiosensitivity of NHEJR-related *Lig4*-KO cells and B02 had no effect on radiosensitivity of HRR-related *Rad54*-KO cells in both irradiations. This suggests that the DNA-PKcs inhibitor NU7026 and Rad51 inhibitor B02 sensitized cancer cells to the C ion irradiation via suppression of the NHEJR and HRR pathways, respectively. In contrast, telomeres are specialized DNA-protein structures that cap the ends of a chromatid, protecting the end of the chromosomes from deterioration or from fusion with neighboring chromosomes. Critically short telomeres may results in chromosome aberrations, inducing a DNA damage response or cell death [40, 41]. Consequently, Zhou et al. reported that DNA-PKcs inhibition sensitizes cancer cells to irradiation with C ion irradiation via telomere capping disruption, but not via NHEJR inhibition [42]. This increased sensitivity is due to NU7026 and if the cells of interests had shorter telomeres, even with complete recovery of C ion-induced potentially lethal damage within 24 h of incubation after irradiation, DNA-PKcs inhibition could continue to lower cell survival [42]. Moreover, suppression of DNA-PKcs by small interfering RNA (siRNA) sensitizes cells to x-rays through disruption of mitotic progression after irradiation, without affecting DSB repair [43]. The difference in radiosensitizing mechanism between DSB repair suppression and telomere capping disruption may be explained through mitochondrial DNA damages repaired within 24 h after the irradiation [42], which did not include nucleus DNA damages. It was not possible to confirm if DNA damages in the nucleus have been repaired. Furthermore, the difference in radiosensitizing mechanism between DNA-PKcs inhibitor and siRNA effects are likely due to inhibition of the kinase activity by inhibitor blocking of the DSB repair in both non-dividing and dividing cells, though siRNA mainly affects dividing cells [43].

Targeting NHEJR may be of greater clinical risk than targeting HRR during radiation therapy [15] and may also increase risks of immunosuppression as the NHEJR pathway is an integral part of the V(D)J immune recombinational pathway [44, 45]. Nevertheless, C ion radiotherapy in combination with a low concentration of a NHEJR inhibitor offers superior dose distribution and shorter total treatment time (early stage NSCLC is just 4 fractions, in a week) compared with traditional photon radiotherapy [3]. Furthermore, the suppression of error-prone NHEJR by NHEJR inhibitors may reduce the risk of secondary cancers after radiation therapy due to multiple DSBs generated in cells with an intact NHEJR pathway giving rise to high frequencies of misjoining, genomic rearrangements, chromosomal translocations, and genetic instability [46–48]. In addition, cancer stem cells (CSC) are a major cause of recurrence, metastasis, and resistance to radiation [49–51]. The repair of DNA damage in dormant CSC occurs predominantly through the NHEJR pathway but not HRR pathway [52]. Therefore, complete recovery may be expected through combination of NHEJR inhibitors and C ion radiotherapy to kill the CSC.

## Conclusions

Our findings demonstrated that although the sensitizing effect of NHEJR inhibitor to C ion radiotherapy was slightly lower compared with traditional x-ray radiotherapy, NHEJR inhibition increases radiosensitivity of human tumor cells to irradiation with C ions more efficiently relative to HRR inhibition. This also supports the hypothesis that NHEJR is the dominant repair pathway in high-LET radiation as well as in low-LET radiation and offers new treatment strategies in C ion radiotherapy.

## Additional files

Additional file 1: Figure S1. Survival curves after radiation exposure with NU7026 or B02 treatment in human glioblastoma cells. A172 cells (**A** and **B**), U251MG cells (**C** and **D**). NU7026 treatment (**A** and **C**), B02 treatment (**B** and **D**). The presented results are the mean and SD of three independent experiments. Data were statistically evaluated with student’s *t* test with comparisons between inhibitor alone and other treated groups (* *p* < 0.05; ** *p* < 0.01; *** *p* < 0.001). (PDF 122 kb) Additional file 2: Figure S2. Radiosensitization with NU7026 or B02 treatment in human glioblastoma cells. Survival curves (**A** and **B**), Sensitization ratio values (**C** and **D**). A172 cells (**A** and **C**), U251MG cells (**B** and **D**) after exposure to C ion irradiation. The presented results are the mean and SD of three independent experiments. Data were statistically evaluated with the student’s *t* test with comparisons between irradiation alone and other treated groups (* *p* < 0.05; ** *p* < 0.01). (PDF 119 kb)

## Abbreviations

ATM: Ataxia telangiectasia mutated
C: Carbon
CSC: Cancer stem cells
DMSO: Dimethyl sulfoxide
DNA-PK: DNA-dependent protein kinase
DNA-PKcs: Catalytic subunit of DNA-dependent protein kinase
DSBs: Double-strand breaks
HRR: Homologous recombination repair
LET: Linear energy transfer
MEF: Mouse embryonic fibroblast
MRN: Mre11-Rad50-Nbs1
NHEJR: Non-homologous end-joining repair
NSCLC: Non-small cell lung cancer
PBS: Phosphate-buffered saline
PE: Plating efficiency
RBE: Relative biological effectiveness
SD: Standard deviation
SF: Surviving fractions
SOBP: Spread-out Bragg peak
SR: Sensitization ratio

## References

[1] Schulz-Ertner D, Tsujii H (2007). Particle radiation therapy using proton and heavier ion beams. J Clin Oncol.

[2] Durante M, Loeffler JS (2010). Charged particles in radiation oncology. Nat Rev Clin Oncol.

[3] Tsujii H, Kamada T (2012). A review of update clinical results of carbon ion radiotherapy. Jpn J Clin Oncol.

[4] Ohno T (2013). Particle radiotherapy with carbon ion beams. EPMA J.

[5] Ohnishi T, Mori E, Takahashi A (2009). DNA double-strand breaks: Their production, recognition, and repair in eukaryotes. Mutat Res.

[6] Falk M, Lukasova E, Kozubek S (2010). Higher-order chromatin structure in DSB induction, repair and misrepair. Mutat Res.

[7] Chapman JR, Taylor MR, Boulton SJ (2012). Playing the end game: DNA double-strand break repair pathway choice. Mol Cell.

[8] Wang C, Lees-Miller SP (2013). Detection and repair of ionizing radiation-induced DNA double strand breaks: New developments in nonhomologous end joining. Int J Radiat Oncol Biol Phys.

[9] Jeggo PA, Geuting V, Löbrich M (2011). The role of homologous recombination in radiation-induced double-strand break repair. Radiother Oncol.

[10] Terato H, Tanaka R, Nakaarai Y, Nohara T, Doi Y, Iwai S (2008). Quantitative analysis of isolated and clustered DNA damage induced by gamma-rays, carbon ion beams, and iron ion beams. J Radiat Res.

[11] Asaithamby A, Hu B, Chen DJ (2011). Unrepaired clustered DNA lesions induce chromosome breakage in human cells. Proc Natl Acad Sci U S A.

[12] Okayasu R, Okada M, Okabe A, Noguchi M, Takakura K, Takahashi S (2006). Repair of DNA damage induced by accelerated heavy ions in mammalian cells proficient and deficient in the non-homologous end-joining pathway. Radiat Res.

[13] Takahashi A, Yamakawa N, Kirita T, Omori K, Ishioka N, Furusawa Y (2008). DNA damage recognition proteins localize along heavy ion induced tracks in the cell nucleus. J Radiat Res.

[14] Hall EJ, Giaccia AJ (2012). Radiobiology for the Radiologist.

[15] Wouters BG, Begg AC, Joiner M, van der Kogel A (2009). Irradiation-induced damage and the DNA damage response. Basic Clinical Radiobiology.

[16] Beucher A, Birraux J, Tchouandong L, Barton O, Shibata A, Conrad S (2009). ATM and Artemis promote homologous recombination of radiation-induced DNA double-strand breaks in G_2_. EMBO J.

[17] Shibata A, Conrad S, Birraux J, Geuting V, Barton O, Ismail A (2011). Factors determining DNA double-strand break repair pathway choice in G_2_ phase. EMBO J.

[18] Collis SJ, DeWeese TL, Jeggo PA, Parker AR (2005). The life and death of DNA-PK. Oncogene.

[19] Chan DW, Chen BP, Prithivirajsingh S, Kurimasa A, Story MD, Qin J (2002). Autophosphorylation of the DNA-dependent protein kinase catalytic subunit is required for rejoining of DNA double-strand breaks. Genes Dev.

[20] Chen BP, Chan DW, Kobayashi J, Burma S, Asaithamby A, Morotomi-Yano K (2005). Cell cycle dependence of DNA-dependent protein kinase phosphorylation in response to DNA double strand breaks. J Biol Chem.

[21] Chen BP, Uematsu N, Kobayashi J, Lerenthal Y, Krempler A, Yajima H (2007). Ataxia telangiectasia mutated (ATM) is essential for DNA-PKcs phosphorylations at the Thr-2609 cluster upon DNA double strand break. J Biol Chem.

[22] Zhao Y, Thomas HD, Batey MA, Cowell IG, Richardson CJ, Griffin RJ (2006). Preclinical evaluation of a potent novel DNA-dependent protein kinase inhibitor NU7441. Cancer Res.

[23] Ciszewski WM, Tavecchio M, Dastych J, Curtin NJ (2014). DNA-PK inhibition by NU7441 sensitizes breast cancer cells to ionizing radiation and doxorubicin. Breast Cancer Res Treat.

[24] Shaheen FS, Znojek P, Fisher A, Webster M, Plummer R, Gaughan L (2011). Targeting the DNA double strand break repair machinery in prostate cancer. PLoS One.

[25] Takahashi A, Kubo M, Ma H, Nakagawa A, Yoshida Y, Isono M (2014). Nonhomologous end-joining repair plays a more important role than homologous recombination repair in defining radiosensitivity after exposure to high-LET radiation. Radiat Res.

[26] Ohno T, Kanai T, Yamada S, Yusa K, Tashiro M, Shimada H (2011). Carbon ion radiotherapy at the Gunma University Heavy Ion Medical Center: new facility set-up. Cancers.

[27] Bolderson E, Richard DJ, Zhou BB, Khanna KK (2009). Recent advances in cancer therapy targeting proteins involved in DNA double-strand break repair. Clin Cancer Res.

[28] Gerelchuluun A, Manabe E, Ishikawa T, Sun L, Itoh K, Sakae T (2015). The major DNA repair pathway after both proton and carbon-ion radiation is NHEJ, but the HR pathway is more relevant in carbon ions. Radiat Res.

[29] Yu L, Tumati V, Tseng SF, Hsu FM, Kim DN, Hong D (2012). DAB2IP regulates autophagy in prostate cancer in response to combined treatment of radiation and a DNA-PKcs inhibitor. Neoplasia.

[30] Veuger SJ, Curtin NJ, Richardson CJ, Smith GC, Durkacz BW (2003). Radiosensitization and DNA repair inhibition by the combined use of novel inhibitors of DNA-dependent protein kinase and poly(ADP-ribose) polymerase-1. Cancer Res.

[31] Azad A, Jackson S, Cullinane C, Natoli A, Neilsen PM, Callen DF (2011). Inhibition of DNA-dependent protein kinase induces accelerated senescence in irradiated human cancer cells. Mol Cancer Res.

[32] Li YH, Wang X, Pan Y, Lee DH, Chowdhury D, Kimmelman AC (2012). Inhibition of non-homologous end joining repair impairs pancreatic cancer growth and enhances radiation response. PLoS One.

[33] Ismail IH, Martensson S, Moshinsky D, Rice A, Tang C, Howlett A (2004). SU11752 inhibits the DNA-dependent protein kinase and DNA double-strand break repair resulting in ionizing radiation sensitization. Oncogene.

[34] Fontana AO, Augsburger MA, Grosse N, Guckenberger M, Lomax AJ, Sartori AA (2015). Differential DNA repair pathway choice in cancer cells after proton- and photon-irradiation. Radiother Oncol.

[35] Wang H, Wang X, Zhang P, Wang Y (2008). The Ku-dependent non-homologous end-joining but not other repair pathway is inhibited by high linear energy transfer ionizing radiation. DNA Repair.

[36] Wang H, Zhang X, Wang P, Yu X, Essers J, Chen D (2010). Characteristics of DNA-binding proteins determine the biological sensitivity to high-linear energy transfer radiation. Nucleic Acids Res.

[37] Li Y, Qian H, Wang Y, Cucinotta FA (2012). A stochastic model of DNA fragments rejoining. PLoS One.

[38] Aoki-Nakano M, Furusawa Y (2013). Misrepair of DNA double-strand breaks after exposure to heavy-ion beams causes a peak in the LET-RBE relationship with respect to cell killing in DT40 cells. J Radiat Res.

[39] Jette N, Lees-Miller SP (2015). The DNA-dependent protein kinase: A multifunctional protein kinase with roles in DNA double strand break repair and mitosis. Prog Biophys Mol Biol.

[40] Deng W, Tsao SW, Guan XY, Lucas JN, Si HX, Leung CS (2004). Distinct profiles of critically short telomeres are a key determinant of different chromosome aberrations in immortalized human cells: whole-genome evidence from multiple cell lines. Oncogene.

[41] IJpma AS, Greider CW (2003). Short telomeres induce a DNA damage response in saccharomyces cerevisiae. Mol Biol Cell.

[42] Zhou X, Zhang X, Xie Y, Tanaka K, Wang B, Zhang H (2013). DNA-PKcs inhibition sensitizes cancer cells to carbon-ion irradiation via telomere capping disruption. PLoS One.

[43] Gustafsson AS, Abramenkovs A, Stenerlöw B (2014). Suppression of DNA-dependent protein kinase sensitize cells to radiation without affecting DSB repair. Mutat Res.

[44] Malu S, Malshetty V, Francis D, Cortes P (2012). Role of non-homologous end joining in V(D)J recombination. Immunol Res.

[45] Jung D, Giallourakis C, Mostoslavsky R, Alt FW (2006). Mechanism and control of V(D)J recombination at the immunoglobulin heavy chain locus. Annu Rev Immunol.

[46] Rothkamm K, Kuhne M, Jeggo PA, Lobrich M (2001). Radiation-induced genomic rearrangements formed by nonhomologous end-joining of DNA double-strand breaks. Cancer Res.

[47] Rief N, Lobrich M (2002). Efficient rejoining of radiation-induced DNA double-strand breaks in centromeric DNA of human cells. J Biol Chem.

[48] Elliott B, Jasin M (2002). Double-strand breaks and translocations in cancer. Cell Mol Lif Sci.

[49] Bao S, Wu Q, McLendon RE, Hao Y, Shi Q, Hjelmeland AB (2006). Glioma stem cells promote radioresistance by preferential activation of the DNA damage response. Nature.

[50] Hambardzumyan D, Squatrito M, Holland EC (2006). Radiation resistance and stem-like cells in brain tumors. Cancer Cell.

[51] Rich JN (2007). Cancer stem cells in radiation resistance. Cancer Res.

[52] Nishikawa S, Ishii H, Haraguchi N, Kano Y, Fukusumi T, Ohta K (2012). Genotoxic therapy stimulates error-prone DNA repair in dormant hepatocellular cancer stem cells. Exp Ther Med.

