# Supplementary figures and images for: Combining carbon ion irradiation and non-homologous end-joining repair inhibitor NU7026 efficiently kills cancer cells
Source: Radiat Oncol. 2015 Nov 9;10:225. doi: 10.1186/s13014-015-0536-z (PMC4638098; doi:10.1186/s13014-015-0536-z)

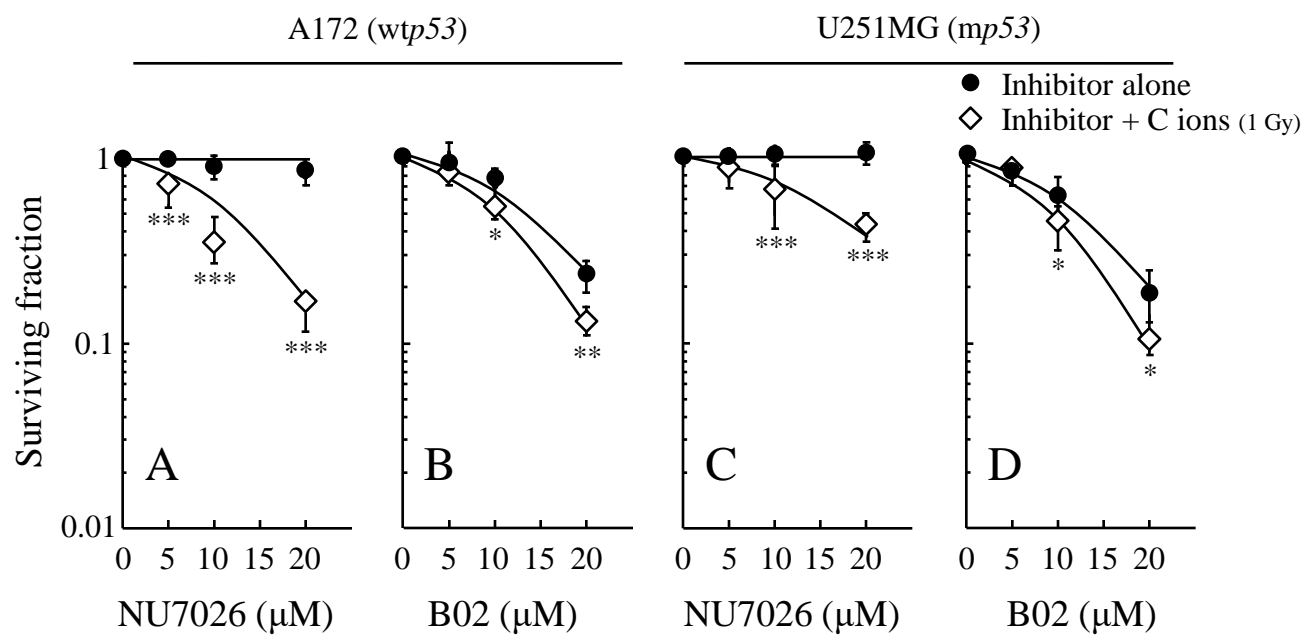

Supplement: Additional file 1: Figure S1. — Survival curves after radiation exposure with NU7026 or B02 treatment in human glioblastoma cells. A172 cells (A and B), U251MG cells (C and D). NU7026 treatment (A and C), B02 treatment (B and D). The presented results are the mean and SD of three independent experiments. Data were statistically evaluated with student’s t test with comparisons between inhibitor alone and other treated groups (* p < 0.05; ** p < 0.01; *** p < 0.001). (PDF 122 kb) [file 13014_2015_536_MOESM1_ESM.pdf]

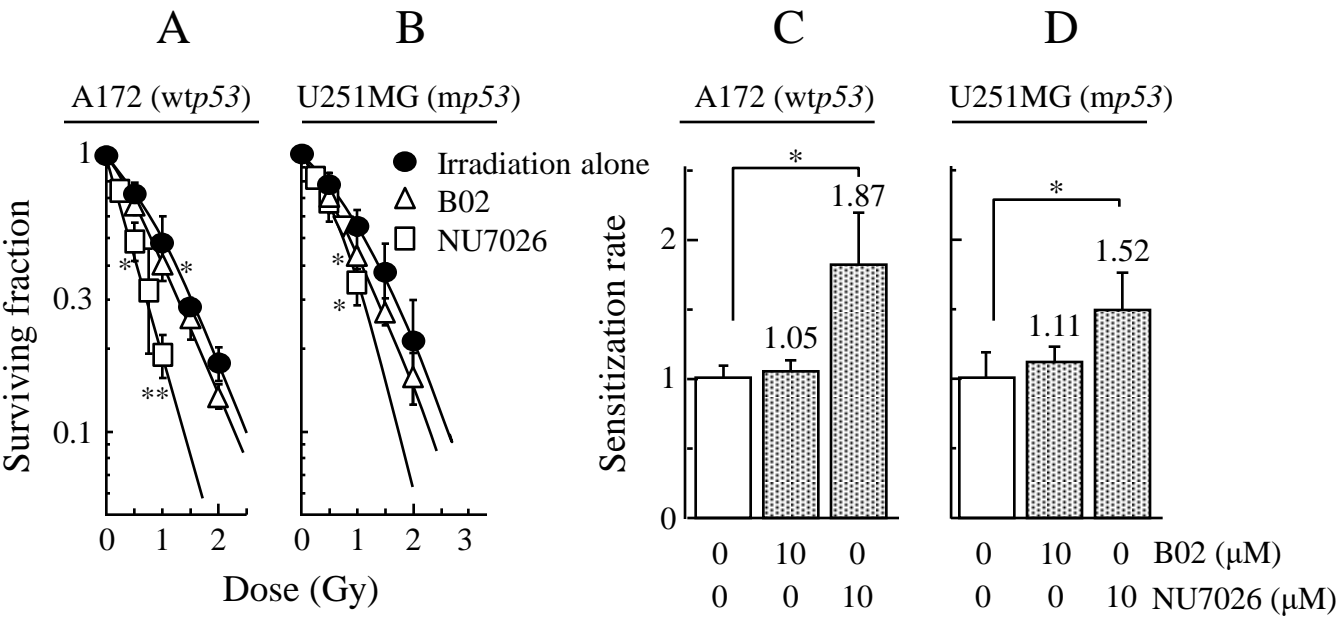

Supplement: Additional file 2: Figure S2. — Radiosensitization with NU7026 or B02 treatment in human glioblastoma cells. Survival curves (A and B), Sensitization ratio values (C and D). A172 cells (A and C), U251MG cells (B and D) after exposure to C ion irradiation. The presented results are the mean and SD of three independent experiments. Data were statistically evaluated with the student’s t test with comparisons between irradiation alone and other treated groups (* p < 0.05; ** p < 0.01). (PDF 119 kb) [file 13014_2015_536_MOESM2_ESM.pdf]
